# Infants With Dorsal Hand Compartment Syndrome Due to Intravenous Infiltration of Glucose Acetate Ringer’s Solution

**DOI:** 10.7759/cureus.54114

**Published:** 2024-02-13

**Authors:** Shinya Tomori, Seigo Korematsu, Satoshi Masutani, Taichi Momose, Yasuko Urushihara, Koichi Moriwaki

**Affiliations:** 1 Pediatrics, Saitama Medical Center, Saitama Medical University, Kawagoe, JPN

**Keywords:** glucose acetate ringer’s solution, extravasation, infusion pump, infiltration, compartment syndrome

## Abstract

Compartment syndrome caused by glucose acetate Ringer’s solution in children has not been sufficiently reported. We report the cases of two children who developed compartment syndrome of the dorsum of the hand and forearm after receiving only glucose acetate Ringer’s solution during hospitalization, with one case requiring a releasing incision. In recent years, glucose acetate Ringer’s solution has been frequently used for maintenance infusion. However, it is not always safe and should be used with caution due to the risk of serious side effects caused by infiltration.

## Introduction

Compartment syndrome of the extremities is a condition in which there is a sudden increase in pressure within a muscle compartment [1]. It can be caused by a variety of factors, including fracture, burn, delivery trauma, and reperfusion, but infusion pump-induced compartment syndrome has not been sufficiently reported, especially in pediatric cases [2].

Here, we report the cases of two children with compartment syndrome that developed during the infusion of glucose acetate Ringer’s solution. One case was treated with a reduced tension incision.

This article was posted to the Research Square preprint server on April 5, 2023.

## Case presentation

Case one

A five-month-old boy was admitted to the hospital for close examination and treatment due to the appearance of black stool on the day before admission.

On admission, the patient was alert. His temperature was 37.9°C, and he had no respiratory disturbance or tachycardia. A physical examination revealed no abnormalities. Contrast-enhanced computed tomography showed a zone of increased blood flow in the right upper abdominal small bowel, which led to the suspicion of Meckel’s diverticulum. After admission, he was started on fasting glucose acetate Ringer’s solution (Na^+^ 130 mEq/L, K^−^ 4 mEq/L, Cl^−^ 109 mEq/L, Ca^2+^ 3 mEq/L, acetate 28 mEq/L, glucose 5.0 g/dL) at 35 mL/hour as replacement fluid. To prevent accidental removal, the puncture sites of the peripheral venous channel and the dorsum of the hand and forearm were not easily visible because they were fixed with tape. At 06:00 on the fourth day of hospitalization, the dorsum of the right hand, which was the puncture site of the peripheral venous system, was swollen, and the needle was removed and re-secured at a different site. The patient was comfortable, and the capillary refilling time was less than two seconds. However, the right radial artery was not palpable, and blister formation was observed. We suspected compartment syndrome and consulted a trauma physician because the pulse of the patient’s right radial artery was not palpable and due to the presence of blister formation. The trauma physician performed intramuscular compartment pressure measurements. The muscle compartment pressure was measured using a previously reported method [3]. We found that the intramuscular pressure in the dorsal interosseous muscles of the hand was elevated to 90 mmHg. We concluded that a decompression incision was necessary.

**Figure 1:**
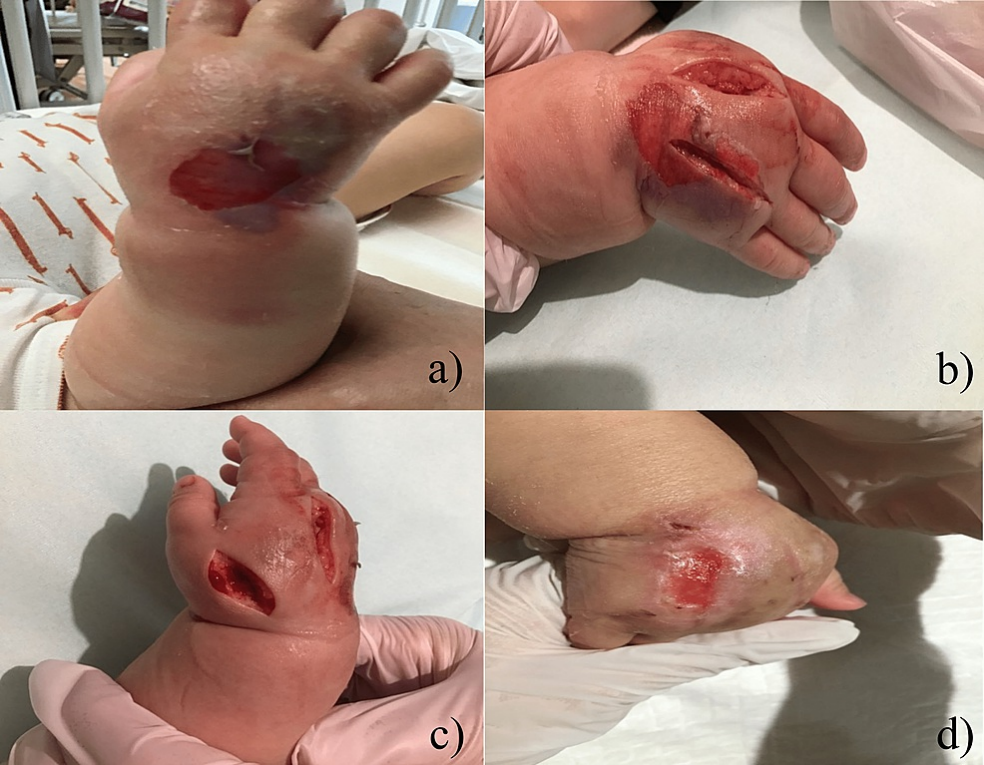
Clinical course of the dorsal lesion of the right hand (Case one). (a) The lesion when the swelling is perceived. Swelling of the dorsum of the hand and a huge blister that has broken off is noted. (b, c) Immediately after releasing the incision. (d) Lesion on day 23 of hospitalization. No contracture was noted at this time.

A release incision was made (Figure 1) and washed daily. The wound was sutured and closed on the sixth day of hospitalization because it was shrinking and its edges were well-defined. The wound was debrided on the 15th day of admission due to necrotic tissue. Thereafter, epithelialization improved with washing, spraying of transferrin, topical antimicrobial treatment, and gauze protection. The patient remained stable throughout his hospital stay and was discharged in good health on the 24th day of admission. Thereafter, there was no functional impairment in the right hand.

Case two

**Figure 2:**
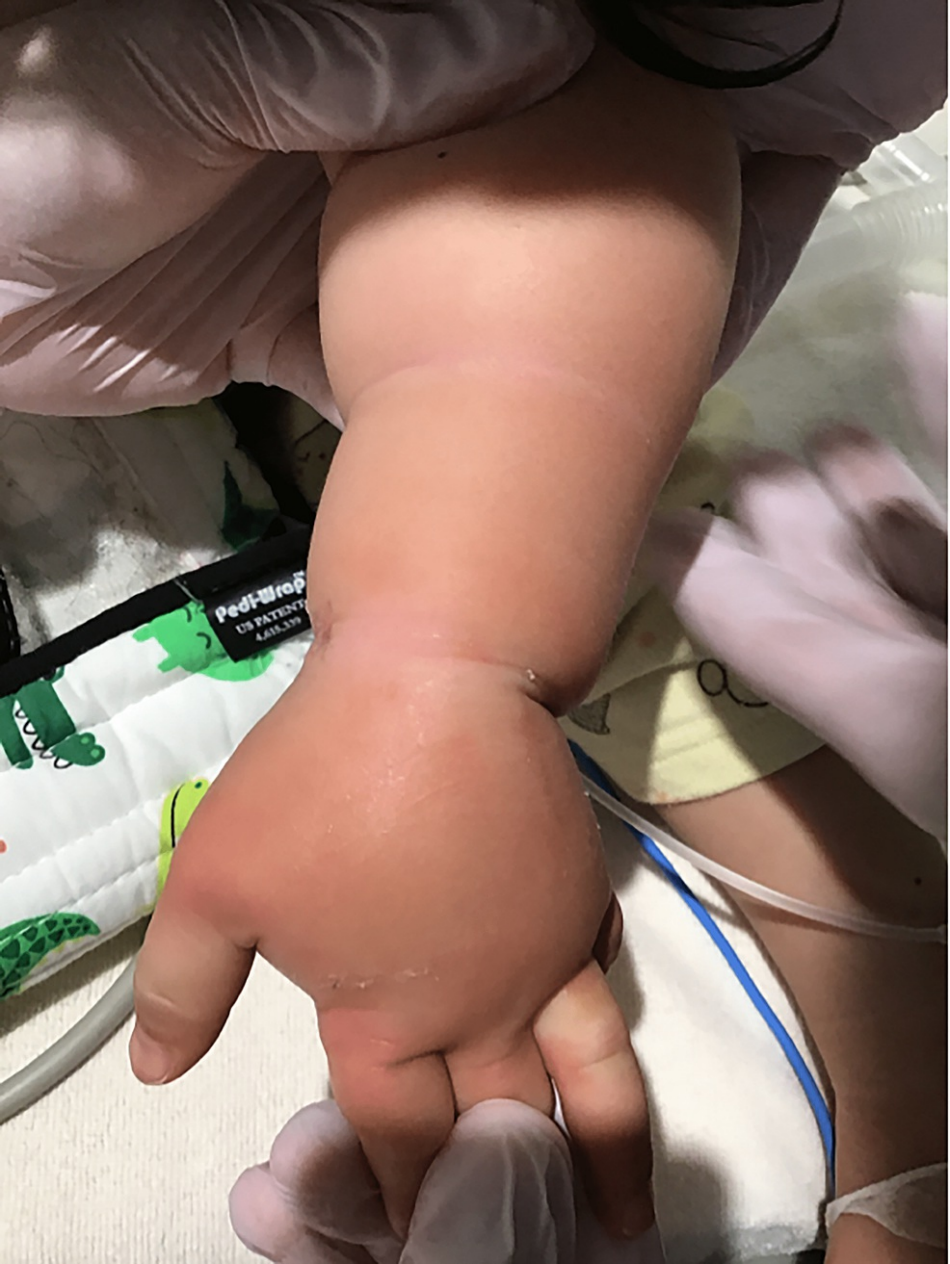
Left dorsal hand two hours after peripheral venous tract extraction (Case two). There was rubor and swelling mainly on the dorsum of her left hand. Her fifth finger was pale.

A one-year-old girl was admitted to the hospital due to febrile convulsions and mild hypoxemia. On admission, she was alert. Her temperature was 40.0°C, and she required oxygen therapy; however, she did not have tachypnea. Her heart rate was 156 beats/minute and regular, but she did not have hypotension. A physical examination revealed no abnormalities. After admission, the patient was managed using the same infusion management as in Case one. At 09:40 on the second day of hospitalization, she complained of pain. We then checked the dorsum of her left hand, which was the site of peripheral venipuncture. We found that her distal left forearm was swollen from the dorsum of the left hand; therefore, the needle was removed and replaced at another location. She was comfortable, and the capillary refilling time was less than two seconds. The patient was able to move her fingers, and the radial artery was palpable. However, she cried strongly when trying to move her hand, and the skin on part of her hand was pale (Figure 2). Therefore, compartment syndrome was suspected, and muscle compartment pressures were measured. Because the measured pressure was less than 30 mmHg at the measurement site, we decided not to perform an emergency decompression incision. SpO_2_ was checked and monitored frequently. In addition, rest and cooling of the dorsum of the left hand and elevation of the left upper limb were performed. By evening, the swelling of the dorsum of her left hand and forearm had improved.

## Discussion

Infiltration of the commonly used glucose acetate Ringer’s solution in both cases caused compartment syndrome of the dorsum of the hand to the forearm, although one case required a reduction incision, and neither case showed permanent functional impairment.

In children, compartment syndrome of the forearm is generally caused by fractures, but a few cases of compartment syndrome caused by infusion or intravenous administration have been reported [2]. The term “infiltration” is defined as the accidental leakage of a non-necrotic agent into surrounding tissues. Extravasation is defined as the accidental leakage of a necrotic agent into surrounding tissues [4]. As fluid passes from the intravenous tubing into the narrow-gauge intravenous catheter, the rate of fluid movement increases exponentially. The high-velocity fluid exiting the tip of the catheter results in high pressure, damaging the venous wall and causing leakage of the drug into the perivascular area [5]. In addition, infiltration or extravasation of fluids or intravenous administration can cause the surrounding tissue pressure to exceed the intracompartmental pressure. This increases venous pressure, which, in turn, further increases the intracompartmental pressure due to increased tissue pressure from the stasis of blood flow [6].

In this report, we present two children with forearm compartment syndrome caused by infiltration or extravasation. We searched PubMed and I-Chu-Shi databases for relevant articles using the search term “infiltration and compartment syndrome” (Table 1).

**Table 1:** Compartment syndrome by intravenous infusion.

Patient	Age	Sex	Reason for admission to the hospital	Contents of infusion	Infusion pump or syringe driver	Location	Releasing incision	Osmotic ratio	Sequelae
Case 1	5 months	Male	Meckel diverticulum	Glucose acetate Ringer’s solution	Used	Right dorsum manus	Performed	1.7	None
Case 2	1 year	Female	Febrile convulsion and acute upper respiratory inflammation	Glucose acetate Ringer’s solution	Used	Left dorsum manus and forearm	None	1.7	None
Ouellette and Kelly (1996) [7]	6 months	Female	Meningitis	Dopamine	Unknown	Dorsum manus and forearm	Performed	0.8	None
	9 years	Female	Cerebral aneurysm	Dilantin	Unknown	Dorsum manus and forearm	Performed	29	None
	5 months	Female	Meningitis	Dilantin	Unknown	Forearm	Performed	29	Below-the-elbow amputation
	1 year	Male	Pneumonia	Aminophylline	Unknown	Dorsum manus and forearm	Unknown	0.4	None
Stahl and Lerner (2000) [8]	10 years	Female	Left femoral subtrochanteric fracture	20% mannitol	Unknown	Left dorsum manus and forearm	Performed	3	None
Spenny et al. (2004) [9]	3 years	Female	RSV bronchiolitis	Methylprednisolone, normal saline, 5% dextrose in half saline CTRX	Unknown	Left dorsum manus	Performed	2 1, 1.4, 1.5	None
Altan et al. (2013) [10]	23 days	Male	pulmonary disease	Iohexol	Used	Right dorsum manus and forearm	Performed	3	None
Chen et al. (2010) [11]	4 days	Male	Hypocalcemia	10% calcium gluconate	Used	Right medial malleolus	Performed	0.9	None
Yamaguchi et al. (2022) [6]	1 year	Female	Endoscope	Glucose acetate Ringer’s solution	Used	Left dorsum manus and forearm	Performed	1.7	None

The median age of the 11 previous cases was six months (range: four months to nine years). Nine of the 11 cases occurred in the forearm or dorsum of the hand. This high prevalence may be because the dorsum of the hand is the most common insertion site for venous catheters in children. The median osmotic ratio in the 11 patients was 1.7 (range: 0.4-29). In addition to compartment syndrome caused by necrotic agents such as vasoactive drugs, contrast media, and drugs with very high osmotic pressure ratios, compartment syndrome may be caused by commonly used infusion products that have low osmotic pressure ratios (e.g., glucose acetate Ringer’s solution). Yamaguchi et al. [6] reported the case of a one-year-old girl who was admitted for examination and received intravenous glucose acetate Ringer’s solution at 30 mL/hour. Every three hours, the nurse checked the puncture site of the peripheral venous channel, but after observing swelling of the entire left superior and poor coloration of the left phalanges, the peripheral venous line was immediately removed. Intra-tissue pressure was measured, a diagnosis of left forearm compartment syndrome was made, and a releasing incision was made. Postoperatively, no residual functional impairment of the left forearm was observed. Spenny et al. [9] reported the case of a three-year-old girl who was admitted with respiratory syncytial virus infection and received 5% dextrose in 0.45% sodium chloride solution at 75 mL/hour via a venous catheter in the dorsum of the left hand. The next morning, blisters were noted on the left elbow just proximal to the intravenous arm board used to secure the catheter. After an infusion of ceftriaxone, the swelling rapidly worsened from the dorsum of the left hand to the forearm, resulting in an emergency fasciotomy for compartment syndrome.

In the past, hypotonic solutions were traditionally used for maintenance infusions in infants; however, with increasing reports of hospital-acquired hyponatremia, the use of isotonic solutions for maintenance infusions in infants is becoming more common worldwide [12]. Although glucose acetate Ringer’s solution is a commonly used preparation in infusion therapy, its osmotic pressure ratio is approximately 2.0 in 0.9% saline. As the osmotic ratio increases, cytotoxicity increases; therefore, an osmotic ratio of 3 and a sugar concentration of 10%-12% are considered acceptable for administration through a peripheral vein [13]. Although glucose acetate Ringer’s solution does not meet this criterion, our cases and previous reports indicate that it must be considered a potential source of infiltration-triggered compartment syndrome (Table 1).

All five cases that stated whether or not they used an infusion pump in Table 1 used an infusion pump. When infiltration occurs, pressurization devices may contribute to the development of compartment syndrome. There are scattered reports of forearm compartment syndrome in adults due to massive infusion under pressure [14,15]. In children, infusion may continue even when tissue pressure increases due to infiltration or exudate development, as drug infusion or intravenous administration is often done under positive pressure with a pressurization device rather than by spontaneous titration [6]. Early detection of infiltration is necessary to prevent progression to compartment syndrome.

If infiltration or exudate is detected, the existing guidelines state (1) stopping the administration of intravenous fluids immediately; (2) disconnecting the intravenous tubing from the device; (3) attempting aspiration of the residual drug from the intravenous device; (4) administering nursing interventions, as indicated; and (5) notifying the physician or advanced practice nurse [4].

One of the causes of infiltration and extravasation in children is the difficulty of observation and palpation around the puncture site due to fixation around the venous catheter insertion site, often to prevent accidental removal of the catheter. Although previous reports have described how to respond after extravascular leakage, there are few reports or statements that take into account the characteristics of fixation in children, and no reports suggest how to detect extravascular leakage immediately. The effective ways to detect extravascular fluid leakage in the early stage need to be explored.

In our two cases, the peripheral venous catheter did not penetrate the vessel wall at the time of the puncture, and it is unlikely that the vessel wall was damaged at the time of the puncture. Therefore, the puncture method itself is not problematic. In children when it is difficult to make an infusion route, a puncture on the dorsum of the hand is routinely performed [16]. The speed of infusion from the catheter may have likely played a role in the damage to the venous wall, as the vessels of the dorsal hand in children are relatively small in diameter. An important point of this paper is that even extracellular solutions that are routinely used, such as glucose acetate Ringer’s solution, can cause infiltration. Therefore, recommendations for early detection and response to infiltration are necessary to prevent progression to compartment syndrome.

## Conclusions

We reported the case of two infants who developed compartment syndrome of the dorsal hand while receiving glucose acetate Ringer’s solution alone, with one of the infants requiring a releasing incision. The hypertonic nature of glucose acetate Ringer’s solution with an osmotic ratio of approximately 2.0 increases the possibility of infiltration. When infiltration or extravasation is detected, immediate action should be taken, such as discontinuation of drug administration, removal of the intravenous line, and aspiration of the drug at the time of removal. The possibility of compartment syndrome should also be considered when a drug is administered under pressure using a continuous infusion pump. Although glucose acetate Ringer’s solution is now commonly used for maintenance infusions, one should be aware of its potential for serious complications due to infiltration.
